# Impressive predictive value of ankle-brachial index for very long-term outcomes in patients with cardiovascular disease: IMPACT-ABI study

**DOI:** 10.1371/journal.pone.0177609

**Published:** 2017-06-15

**Authors:** Takashi Miura, Masatoshi Minamisawa, Yasushi Ueki, Naoyuki Abe, Hitoshi Nishimura, Naoto Hashizume, Tomoaki Mochidome, Mikiko Harada, Yasutaka Oguchi, Koji Yoshie, Wataru Shoin, Tatsuya Saigusa, Soichiro Ebisawa, Hirohiko Motoki, Jun Koyama, Uichi Ikeda, Koichiro Kuwahara

**Affiliations:** Department of Cardiovascular Medicine, Shinshu University School of Medicine, Matsumoto, Japan; The University of Tokyo, JAPAN

## Abstract

**Background:**

The ankle—brachial index (ABI) is a marker of generalized atherosclerosis and is predictive of future cardiovascular events. However, few studies have assessed its relation to long-term future cardiovascular events, especially in patients with borderline ABI. We therefore evaluated the relationship between long-term future cardiovascular events and ABI.

**Methods:**

In the IMPACT-ABI study, a single-center, retrospective cohort study, we enrolled 3131 consecutive patients (67 ± 13 years; 82% male) hospitalized for cardiovascular disease and measured ABI between January 2005 and December 2012. After excluding patients with an ABI > 1.4, the remaining 3056 patients were categorized as having low ABI (≤ 0.9), borderline ABI (0.91–0.99), or normal ABI (1.00–1.40). The primary endpoint was MACE (cardiovascular death, myocardial infarction [MI] and stroke). The secondary endpoints were cardiovascular death, MI, stroke, admission due to heart failure, and major bleeding.

**Results:**

During a 4.8-year mean follow-up period, the incidences of MACE (low vs. borderline vs. normal: 32.9% vs. 25.0% vs. 14.6%, P<0.0001) and cardiovascular death (26.2% vs. 18.7% vs. 8.9%, P<0.0001) differed significantly across ABIs. The incidences of stroke (9.1% vs. 8.6% vs. 4.8%, P<0.0001) and heart failure (25.7% vs. 20.8% vs. 8.9%, P<0.0001) were significantly higher in the low and borderline ABI groups than in the normal ABI group. But the incidences of MI and major bleeding were similar in the borderline and normal ABI groups. The hazard ratios for MACE adjusted for traditional atherosclerosis risk factors were significantly higher in patients with low and borderline ABI than those with normal ABI (HR, 1.93; 95%CI: 1.44–2.59, P < 0.0001, HR, 1.54; 95% CI: 1.03–2.29, P = 0.035).

**Conclusions:**

The incidence of long-term adverse events was markedly higher among patients with low or borderline ABI than among those with normal ABI. This suggests that more attention should be paid to patients with borderline ABIs, especially with regard to cardiovascular death, stroke, and heart failure.

## Introduction

The ankle-brachial index (ABI) is a useful tool for specific, cost-effective, noninvasive diagnosis for peripheral artery disease. [1, 2] Between 9% and 23% of people over 55 years of age are affected by peripheral artery disease [3, 4] Among them, only 10% exhibit the typical symptoms of intermittent claudication, whereas 50% have other leg symptoms and the remaining 40% are asymptomatic. [5, 6] Moreover, a low ABI (≤0.9) is strongly associated with generalized atherosclerosis, cardiovascular mortality and all-cause death, regardless of the presence of symptoms. [7, 8] Recently, the American College of Cardiology and the American Heart Association (ACC/AHA) guidelines for the management of peripheral artery disease patients recommended that 0.91 ≤ ABI ≤ 0.99 be considered borderline, and that patients with borderline ABI should be considered a high risk group, similar to patients with an abnormal ABI (ABI ≤ 0.9). [9, 10] However, there were few data available on the long-term future cardiovascular events in patients with borderline ABI, especially among the hospitalized cardiology population, who are admitted to the hospital most frequently. There is thus a clear necessity for real-world data on the long-term likelihood of future cardiovascular events in patients with ABI ≤ 0.9, especially among the hospitalized cardiology population with borderline ABI. To address that issue, we evaluated the relationship between the long-term future cardiovascular events and ABI among the hospitalized cardiology population.

## Methods

### Study population

**Fig 1:**
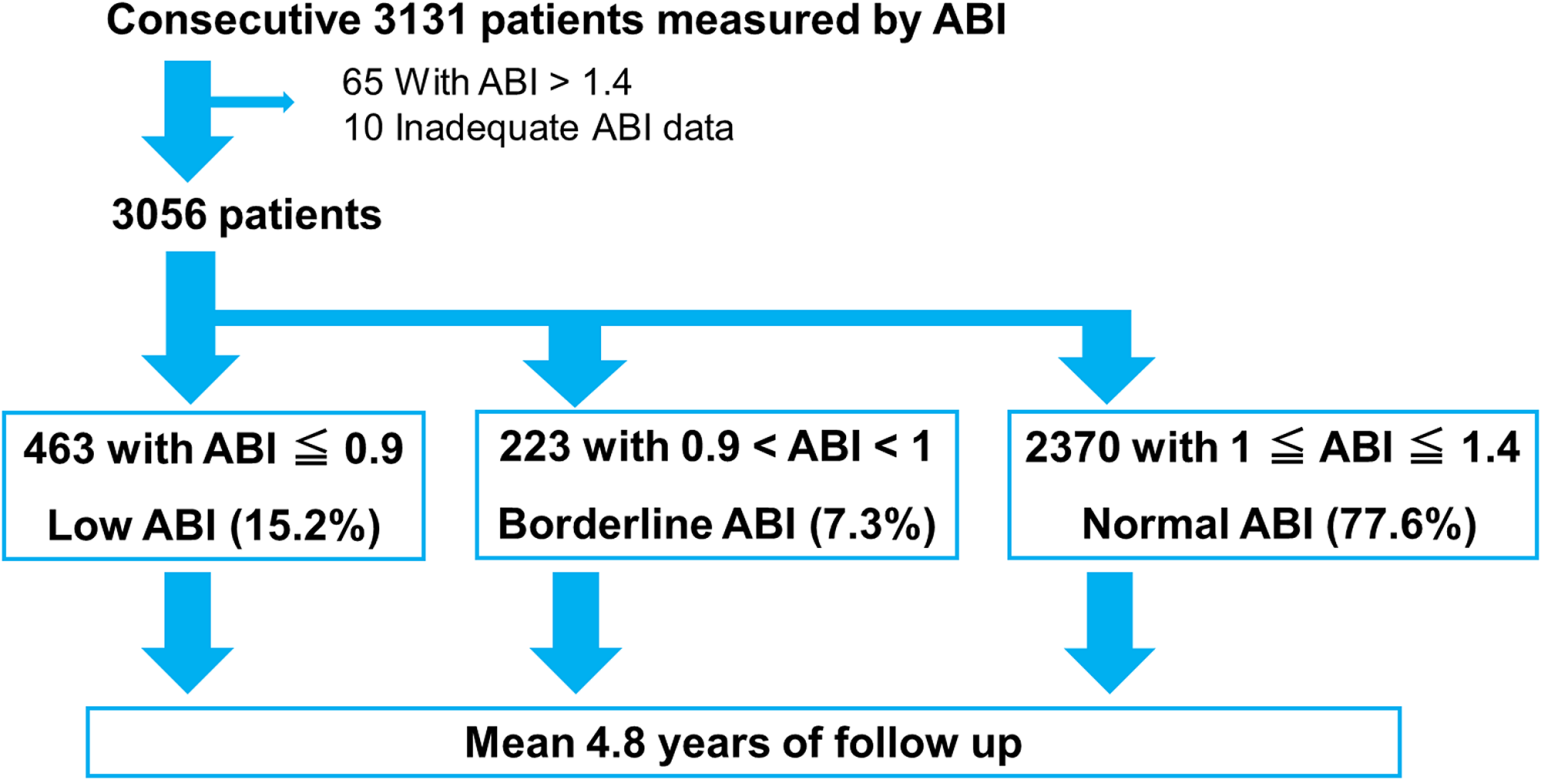
Study flowchart.

The IMPACT-ABI (Impressive Predictive Value of Ankle-Brachial Index for Very Long Term Outcome in Patients with Cardiovascular Disease) study is a single-center, retrospective cohort study. Between January 2005 and December 2012, a total of 3131 consecutive patients were hospitalized for cardiovascular disease and ABI was measured. Ten subjects with inadequate ABI data were excluded, 65 subjects with ABI > 1.4, who were considered to have incompressible calcified arteries in their legs were also excluded. The remaining 3056 patients were categorized into low ABI (≤0.9), borderline ABI (0.9 < ABI < 1.00), and normal ABI (1.00 ≤ ABI ≤ 1.40), groups, based on the ACC/AHA guidelines (Fig 1). [10]

### ABI measurement

Blood pressure measurements and ABI calculations were performed according to AHA recommendations. [11] The ABI was measured with the subject in a supine position after at least 10 minutes of rest using an automatic oscillometric apparatus (Form PWV/ABI; Omron Healthcare, Kyoto, Japan). Four oscillometric cuffs were applied, one to each arm and ankle. The cuffs were connected to a central unit that contained four pressure control pumps and four pressure sensors to automatically measure the blood pressure in the four limbs. The ABI was automatically calculated as the ankle systolic blood pressure (SBP)/brachial SBP ratio using the higher value of the brachial SBP between the right and left arms. Two ABIs were simultaneously measured for the right and left sides, and the lower value was used for analysis.

### Data collection and follow-up

Follow-up data were obtained from hospital charts, by direct contact with patients or from referring physicians. To ensure accurate assessment of clinical events, additional information was obtained from visits or telephone contacts with living patients or from family members, and from medical records obtained from other hospitals, as necessary, between May 2015 and August 2015. Clinical data on 2360 patients (77.2%) were acquired during the research period. The mean follow-up period was 4.8 years±2.7 years (2.8–7.1 years). The primary endpoint was MACE (cardiovascular death, myocardial infarction [MI], and stroke). The secondary endpoints were cardiovascular death, MI, stroke, major bleeding, and heart failure.

### Definitions

Hypertension (HT) was defined as current systolic blood pressure ≥ 140 mmHg and/or diastolic blood pressure ≥ 90 mmHg or use of antihypertensive agents. Dyslipidemia was defined as total cholesterol ≥ 220 mg/dl, low-density lipoprotein cholesterol ≥ 140 mg/dl, high-density lipoprotein cholesterol ≤ 40 mg/dl, triglycerides ≥150 mg/dl, or use of cholesterol-lowering agents. Diabetes mellitus was defined as fasting blood glucose ≥126 mg/dl and/or casual plasma glucose ≥ 200 mg/dl, HbA1c ≥ 6.5% or use of hypoglycemic agents. A smoking habit was defined as previous/current smoking as obtained by interview. Chronic kidney disease was defined as estimated glomerular filtration rate (eGFR) < 60 mL/min/1.73 m^2^.[12] LV dysfunction was defined as left ventricular ejection fraction (LVEF) ≤ 40%.

The requirement of written informed consent was waived by the hospital ethics committee because this study used retrospective data obtained mainly from the hospital records. And these data were fully anonymized before access by the researchers. The protocol for this study was approved by the hospital ethics committee, and the study was performed in accordance with the Declaration of Helsinki. Ethics committee approval was obtained from the Committee for Medical Ethics of Shinshu University School of Medicine (3343). The IMPACT-ABI study is registered with the University Hospital Medical Information Network-Clinical Trials Registry (UMIN-CTR), as accepted by the International Committee of Medical Journal Editors (No. UMIN 000020276).

### Statistical analysis

Continuous variables are reported as the mean ± standard deviation. Categorical variables are reported as frequencies and percentages. Characteristics of the patients in the three groups were compared using the chi-squared test. Continuous variables were compared using analysis of variance. Cumulative frequencies were estimated using the Kaplan-Meier method, and differences were evaluated with the Log-rank test. For each group, multivariate Cox proportional hazard models were used to adjust for the effects of baseline risk factors on major adverse cardiovascular events. ABI value was adjusted by variables considered as independent predictors in the univariate COX regression analysis without strong correlation with other variables. Therefore, BNP, LV dysfunction, chronic kidney disease, diabetes, warfarin administration, and beta-blocker administration were not used as adjusting variables on multivariate Cox regression analysis. P < 0.05 was considered to indicate a statistically significant difference. Statistical analyses were performed using IBM SPSS Statistics Version 21 software.

## Results

### Baseline demographics

Table 1 presents the baseline characteristics of our IMPACT-ABI study participants subdivided based on their ABI categories. Among the 3056 patients, 2370 (77.6%) had a normal ABI, 223 (7.3%) had a borderline ABI, and 463 (15.2%) had a low ABI. There were marked differences between the groups with respect to age, gender, body mass index, hypertension, diabetes mellitus, smoker, hemoglobin, eGFR, hemodialysis, previous stroke, previous heart failure, and left ventricular ejection fraction (LVEF). Age, hypertension, diabetes, smoker, hemodialysis, and previous stroke all increased with decreasing ABI, whereas hemoglobin and eGFR decreased with decreasing ABI. Interestingly, previous heart failure was highest in the borderline ABI group, and LVEF was lowest in the borderline ABI group. Regarding medication, the administration rate of antiplatelet therapies such as aspirin, thienopyridines, and cilostazol increased with decreasing ABI. The causes of admission to hospital are also shown.

**Table 1:** Baseline characteristics stratified based on ABI categories.

variables	ABI ≤ 0.90	0.91 ≤ ABI ≤ 0.99	1.0 ≤ ABI ≤ 1.4	p-value
	N = 463	N = 223	N = 2370	
Age (yrs)	72.2±11.4	68.0±15.2	65.9±13.3	<0.0001
Female	88 (19.0%)	68 (30.5%)	702 (29.6%)	<0.0001
Body mass index (kg/m^2^)	22.8±3.7	23.1±4.1	23.6±3.7	<0.0001
Hypertension	349 (75.3%)	138 (61.9%)	1365 (57.6%)	<0.0001
Dyslipidemia	190 (41.0%)	87 (39.0%)	1025 (43.3%)	0.386
Diabetes mellitus	202 (43.6%)	65 (29.2%)	601 (25.4%)	<0.0001
Insulin use	29 (6.3%)	9 (4.0%)	81 (3.4%)	0.015
Smoker	273 (59.0%)	97 (43.5%)	1028 (43.4%)	<0.0001
Hemoglobin (g/dl)	13.1±2.2	13.7±2.0	14.0±3.2	<0.0001
eGFR (ml/min/1.73m^2^)	48.5±27.4	56.0±25.4	64.5±22.9	<0.0001
Hemodialysis	66 (14.3%)	14 (6.3%)	76 (3.2%)	<0.0001
Previous MI (%)	67 (14.5%)	33 (14.8%)	335 (14.1%)	0.944
Previous stroke (%)	72 (15.6%)	22 (9.9%)	136 (5.7%)	<0.0001
Previous Heart failure (%)	47 (10.2%)	26 (11.7%)	159 (6.7%)	0.002
LVEF (%)	64.0±14.6	61.0±16.5	65.1±14.4	<0.0001
Atrial fibrillation (%)	65 (14.0%)	36 (16.1%)	290 (12.2%)	0.167
CHADS_2_	2.09±1.18	1.68±1.19	1.30±1.08	<0.0001
CHA_2_DS_2_-VASc	3.28±1.35	2.56±1.39	2.11±1.26	<0.0001
baPWV	2013±937	1900±698	1706±437	<0.0001
** Medication at discharge**				
Aspirin	270 (58.3%)	103 (46.2%)	995 (42.0%)	<0.0001
Thienopyridines	145 (31.3%)	52 (23.3%)	536 (22.6%)	<0.0001
Cilostazol	126 (27.2%)	19 (8.5%)	70 (3.0%)	<0.0001
Statins	179 (38.7%)	89 (39.9%)	907 (38.3%)	0.879
ACE-Is/ARBs	261 (56.4%)	105 (47.1%)	1070 (45.2%)	<0.0001
Beta-blockers	111 (24.0%)	63 (28.3%)	633 (26.7%)	0.362
Anti-coagulants	103 (22.2%)	59 (26.5%)	462 (19.5%)	0.366
** Cause of admission**				
Ischemic heart disease	230 (49.7%)	118 (52.9%)	1339 (56.5%)	0.031
Peripheral artery disease	200 (43.2%)	16 (7.2%)	37 (15.6%)	<0.0001
Arrhythmia	23 (5.0%)	42 (18.8%)	519 (21.9%)	<0.0001
Congestive heart failure	42 (9.1%)	46 (20.6%)	347 (14.6%)	0.001

HR, hazard ratio; CI, confidence interval; ABI, ankle—brachial index; eGFR, Estimated glomerular filtration rate; LVEF, Left ventricular ejection fraction; baPWV, brachial-ankle pulse wave velocity; ACE-I, angiotensin-converting-enzyme inhibitor; ARB, Angiotensin II Receptor Blocker.

Data are shown as the mean ± SD or as n (percentages).

### Clinical outcomes in patients separated based on ABI category

**Fig 2:**
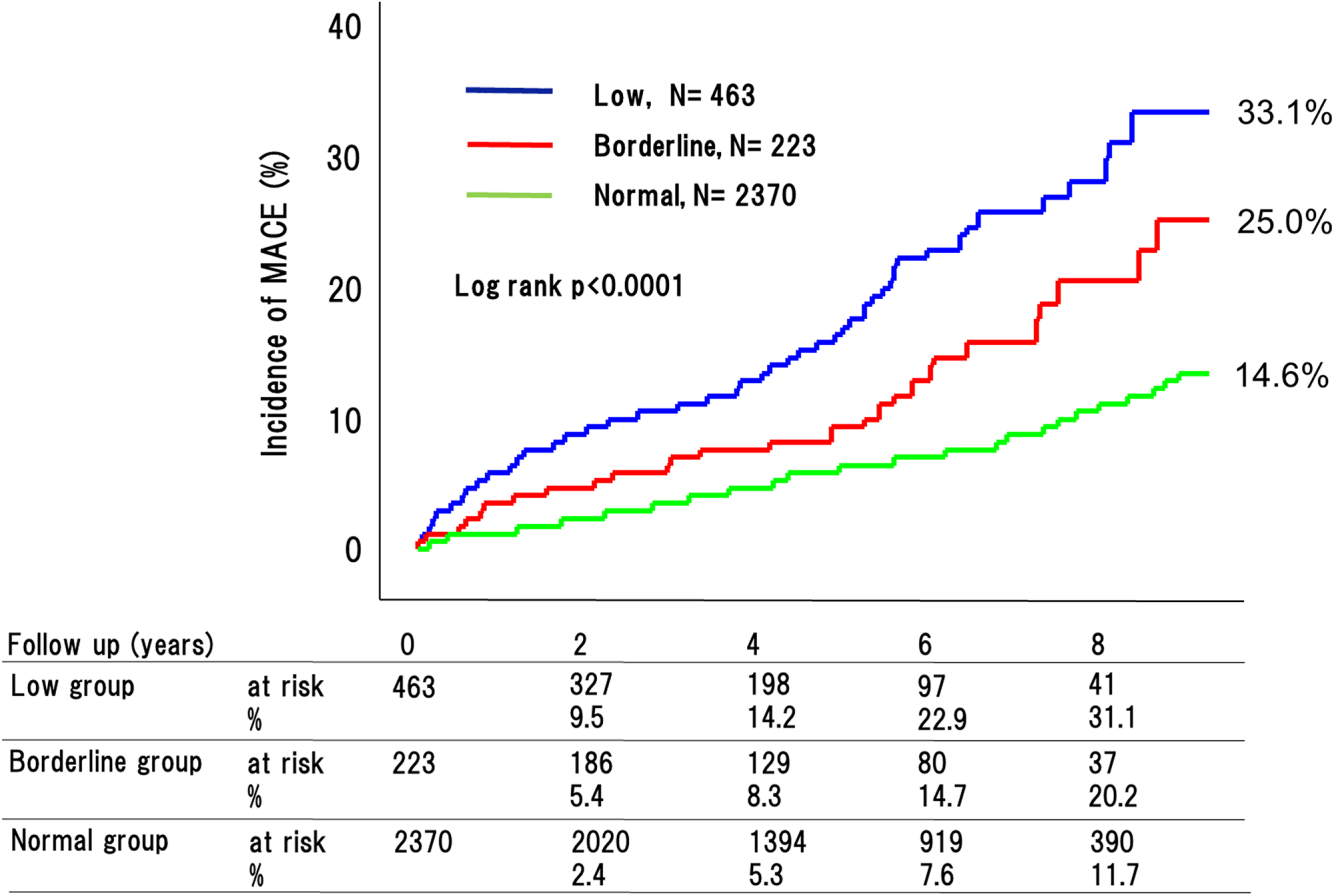
Incidence of major adverse cardiovascular events in the low, borderline and normal ABI groups. The incidences of major adverse cardiovascular events (MACE; includes cardiovascular death, myocardial infarction [MI], and stroke) significantly increased with decreasing ABI.

**Fig 3:**
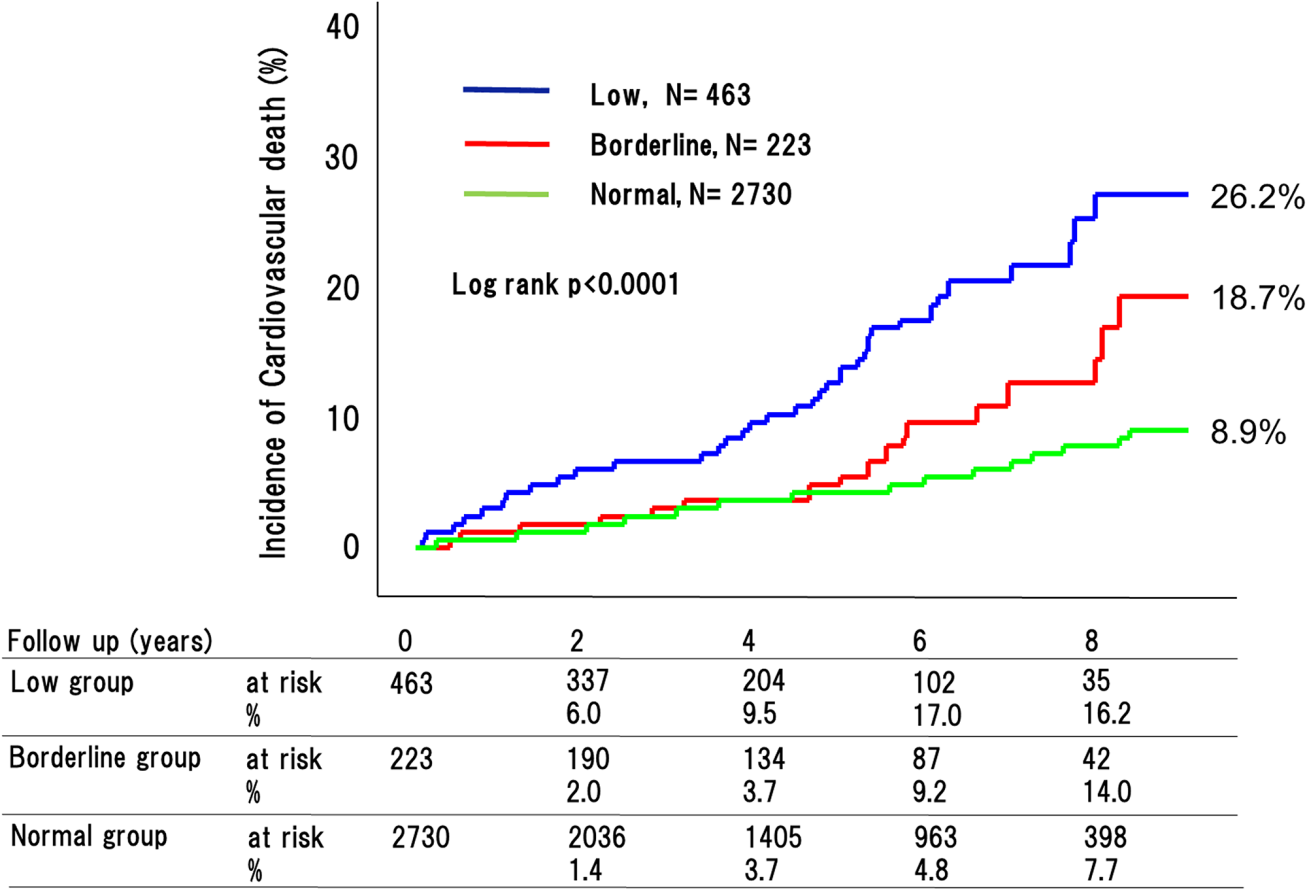
Incidence of cardiovascular death in the low, borderline and normal ABI groups. The incidences of cardiovascular death significantly increased with decreasing ABI.

**Fig 4:**
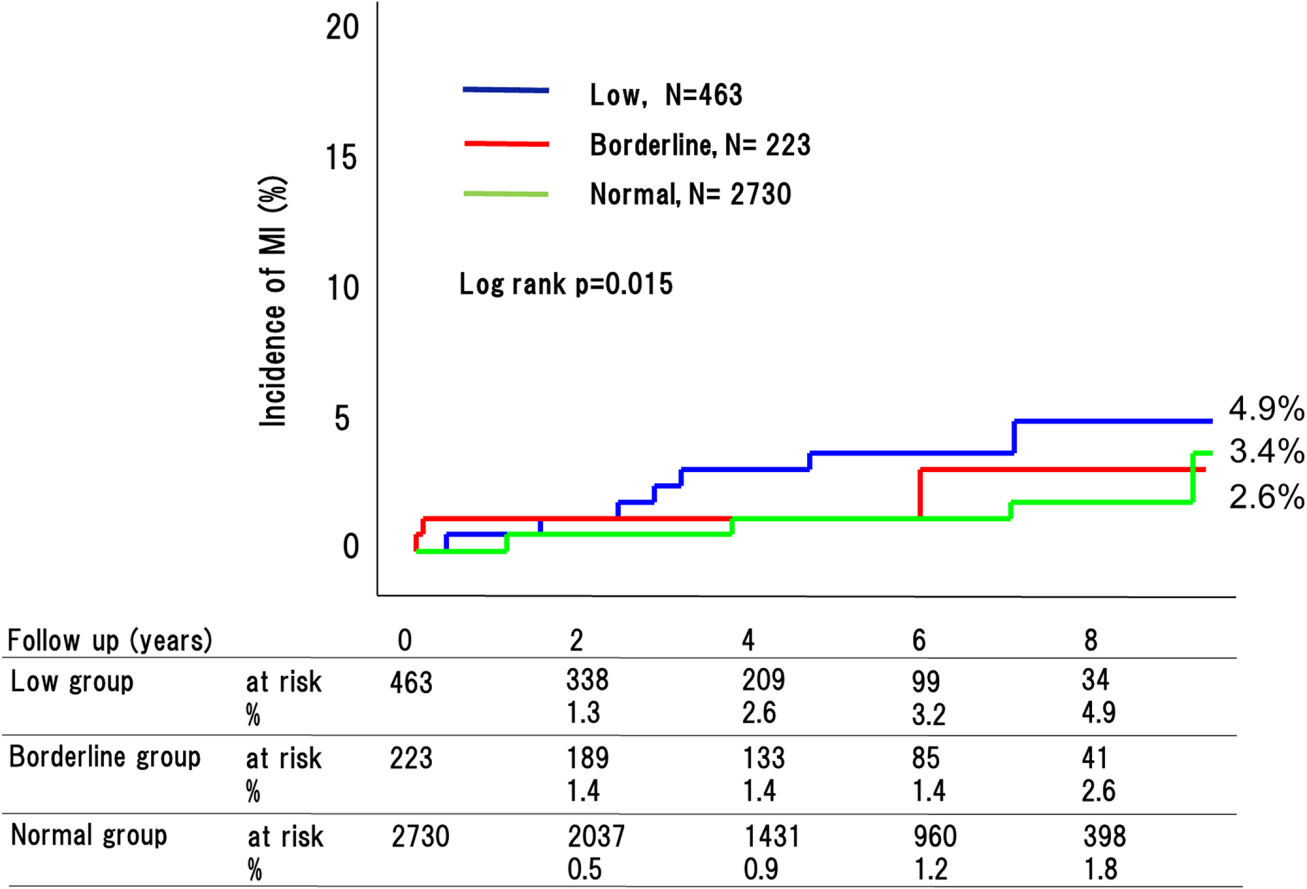
Incidence of MI in the low, borderline and normal ABI groups. The incidences of MI differed greatly among the three groups.

**Fig 5:**
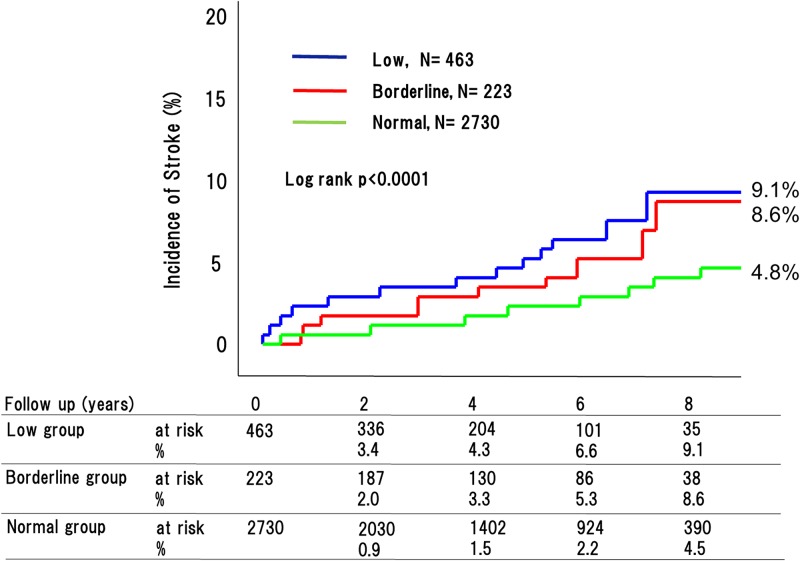
Incidence of stroke in the low, borderline and normal ABI groups. The incidences of stroke in the low and borderline ABI groups were much higher than in the normal ABI group.

**Fig 6:**
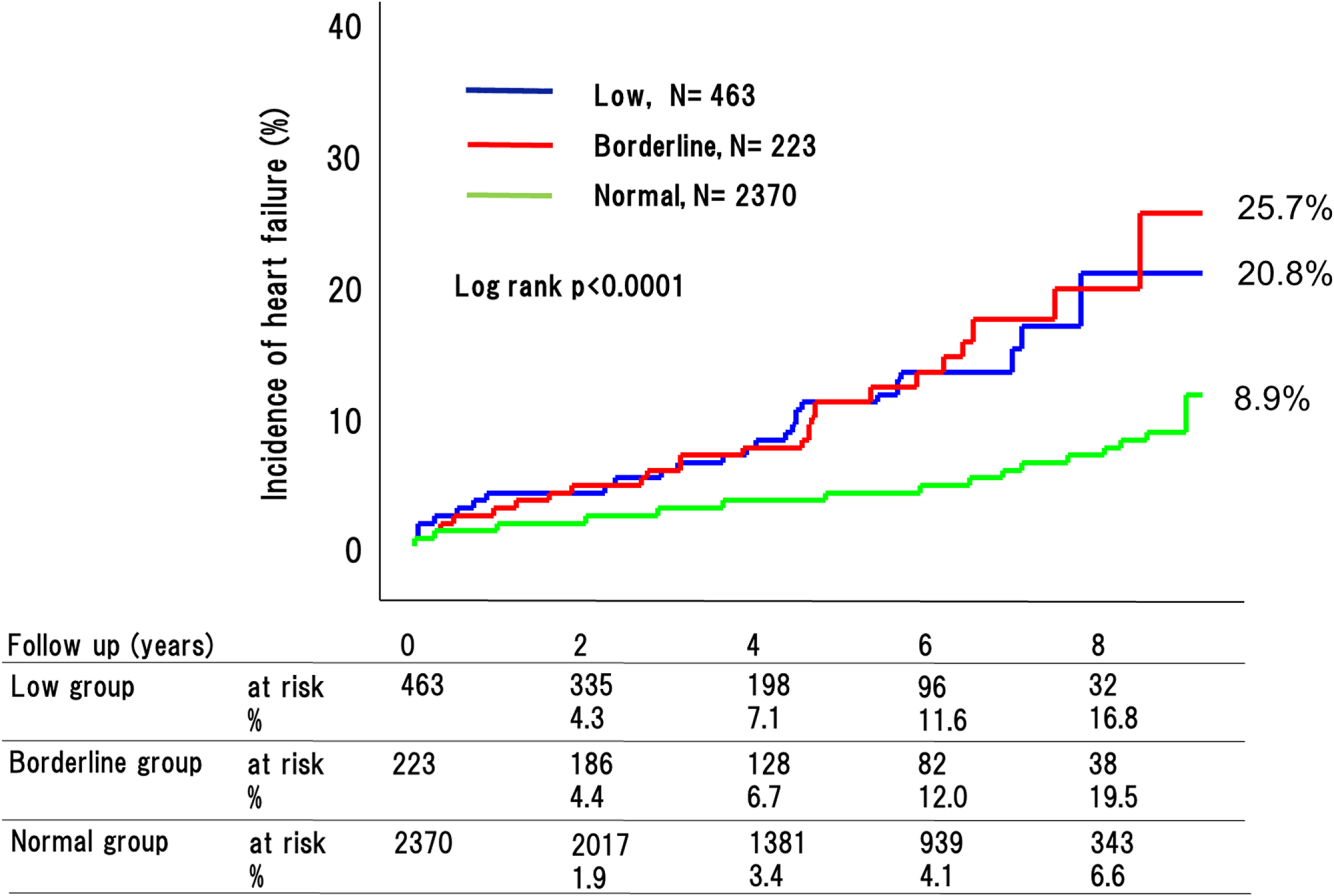
Incidence of heart failure in the low, borderline and normal ABI groups. The incidences of heart failure in the low and borderline ABI groups were much higher than in the normal ABI group.

**Fig 7:**
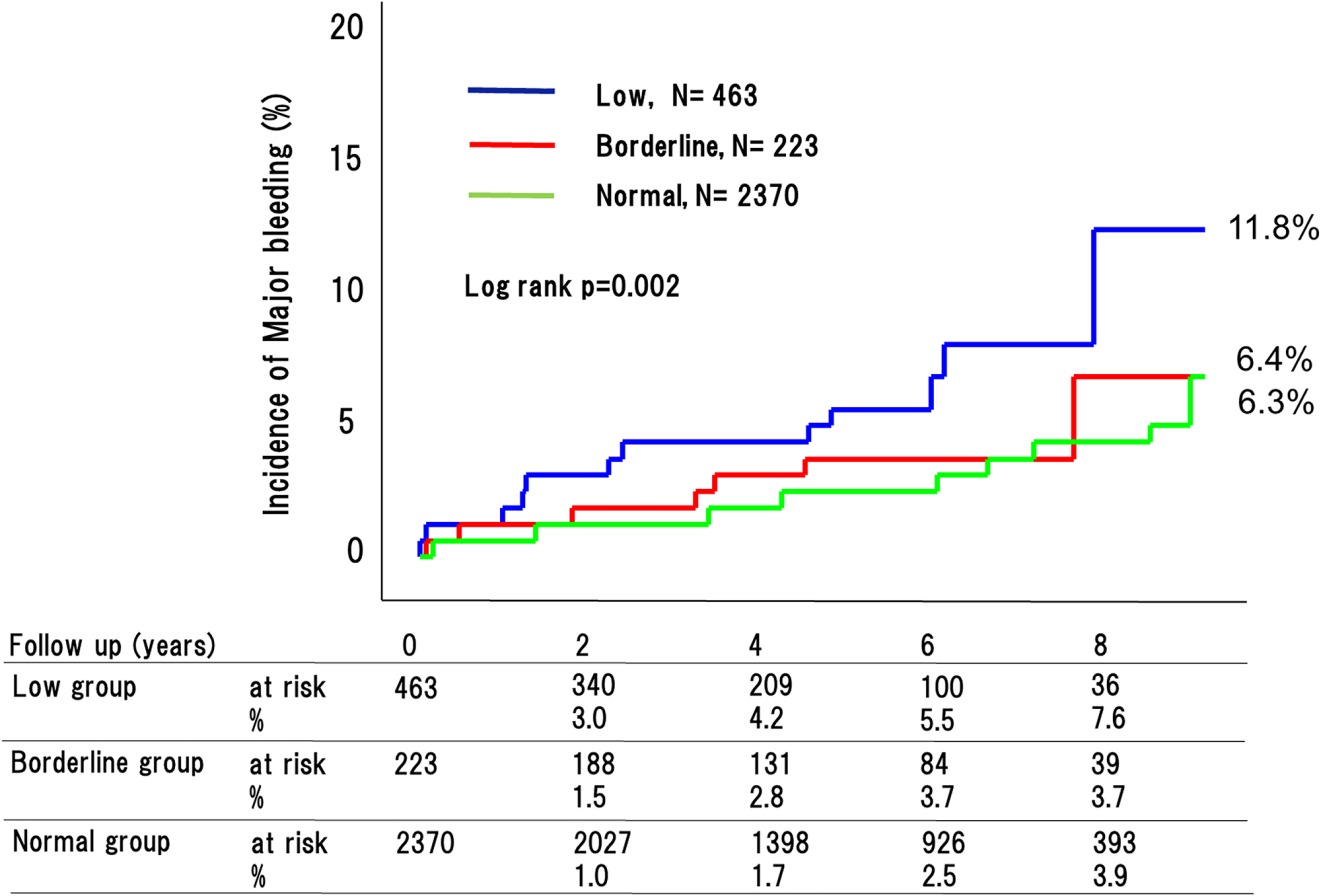
Incidence of major bleeding in the low, borderline and normal ABI groups. The incidences of major bleeding differed greatly among the three groups.

During the 4.8-year mean follow up period, 270 adverse cardiovascular events occurred (76 [32.9%] in patients with low ABI, 29 [20.2%] with borderline ABI, and 165 [11.7%] with normal ABI [P < 0.0001]) (Fig 2). This included 180 cardiovascular deaths (53 [26.2%] in patients with low ABI, 18 [18.7%] with borderline ABI, and 109 [8.9%] with normal ABI [P < 0.0001]) (Fig 3); 90 strokes (22 [9.1%] in patients with low ABI, 10 [8.6%] with borderline ABI, and 58 [4.8%] with normal ABI [P < 0.0001]) (Fig 4); and 41 MIs (11 [4.9%] in patients with low ABI, 4 [2.6%] with borderline ABI, and 26 [3.4%] with normal ABI [P = 0.015]) (Fig 5). The incidence of adverse cardiovascular events increased significantly with decreasing ABI. In terms of other endpoints, there were 164 occurrences of heart failure (39 [25.7%] in patients with low ABI, 25 [20.8%] with borderline ABI, and 100 [8.9%] with normal ABI [P < 0.0001]) (Fig 6) and 85 occurrences of major bleeding (57 [11.8%] in patients with low ABI, 8 [6.4%] with borderline ABI, and 20 [6.3%] with normal ABI [P = 0.002]) (Fig 7).

Multivariate Cox proportional hazards analysis was performed to evaluate the effects of ABI and other predictors on MACE. We found that both low ABI (HR, 1.93; 95% CI: 1.44–2.59, P < 0.001) and borderline ABI (HR, 1.54; 95% CI: 1.03–2.29, P = 0.035) were predictive of adverse cardiovascular events after adjustment for other independent predictors (Table 2).

**Table 2:** Cox proportional regression analysis of MACE in the overall Cox regression analysis.

Variable	unadjusted HR	95% CI	p-value	adjusted HR	95% CI	p-value
Age	1.04	1.03–1.06	<0.0001	1.04	1.03–1.05	<0.0001
Female gender	0.71	0.53–0.94	0.018	0.79	0.57–1.10	0.157
ABI ≤ 0.9	2.92	2.24–3.81	<0.0001	1.94	1.45–2.61	<0.0001
0.91 ≤ ABI ≤ 0.99	1.52	1.03–2.23	0.034	1.53	1.03–2.27	0.035
Body mass index	0.96	0.93–0.99	0.025	0.98	0.95–1.02	0.31
Hb	0.87	0.82–0.92	<0.0001	0.96	0.90–1.03	0.274
UA	0.99	0.94–1.04	0.648			
BNP	1.001	1.00–1.001	<0.0001			
Previous HF	2.84	2.06–3.91	<0.0001	2.69	1.91–3.77	<0.0001
LV dysfunction	2.21	1.68–2.92	<0.0001			
Previous MI	1.49	1.10–2.01	0.01	1.41	1.04–1.93	0.03
Previous stroke	2.26	1.61–3.17	<0.0001	1.63	1.15–2.32	0.007
Previous Intra cranial bleeding	1.46	0.60–3.53	0.403			
Hemodialysis	3.75	2.59–5.44	<0.0001	3.18	2.11–4.81	<0.0001
CKD	2.5	1.96–3.18	<0.0001			
Atrial fibrillation	1.44	1.05–1.98	0.026	1.2	0.86–1.67	0.297
Hypertension	1.32	1.02–1.70	0.033	0.9	0.69–1.18	0.448
Dyslipidemia	0.75	0.59–0.96	0.024	0.73	0.57–0.95	0.02
Smoking	1.34	1.05–1.70	0.017	1.23	0.94–1.62	0.13
Diabetes	1.43	1.12–1.84	0.005			
Insulin user	2.01	1.25–3.25	0.004	1.65	0.99–2.73	0.051
Aspirins administration	1.18	0.93–1.51	0.181			
Warfarins administration	1.38	1.05–1.82	0.021			
betablockers administration	1.49	1.16–1.93	0.002			

HR, hazard ratio; CI, confidence interval; ABI, ankle—brachial index; UA, uric acid; BNP, brain natriuretic peptide; HF, heart failure; MI, myocardial infarction; CKD, chronic kidney disease.

### Clinical outcomes in patients without hemodialysis separated based on ABI category

Generally, hemodialysis was strong predictor of cardiovascular events. Therefore, we evaluated incidence of future cardiovascular events across the ABI categories in non-hemodialysis patients. Even so, the incidence of adverse cardiovascular events and cardiovascular death increased significantly with decreasing ABI (30.2% vs 22.6% vs 14.1%, P<0.0001, 24.1% vs 16.5% vs 8.6%, P<0.0001, respectively). Multivariate Cox analysis also showed that not only low but also borderline ABI was an independent predictor of adverse cardiovascular events (HR, 1.77; 95% CI: 1.28–2.44, P < 0.0001, HR, 1.66; 95% CI: 1.10–2.51, P = 0.013, respectively) (S1 Table).

## Discussion

Our main new findings were, 1) that hospitalized cardiology patients with a low or borderline ABI had a markedly higher incidence of long-term future adverse cardiovascular events; and 2) that hospitalized cardiology patients with borderline ABI had a significantly higher incidence of stroke and heart failure than those with normal ABI; however, 3) the incidences of major bleeding were similar between hospitalized cardiology patients with borderline or normal ABIs.

Earlier studies of diabetic patients, cardiovascular risk patients, and the general patient population showed that patients with borderline ABI had a poorer prognosis than those with normal ABI [7, 13, 14, 15]. Current study could confirm the same trend in hospitalized cardiology patients. However, these reports didn`t show the details of adverse cardiovascular events, such as stroke, incident heart failure, and major bleeding. Therefore, our study was first report that described relationship between incidence of stroke, heart failure and major bleeding and ABI category including borderline ABI.

Patients with borderline ABI had endothelial dysfunction, which is considered as early maker of atherosclerosis [16]. The healthy endothelium maintains vascular tone and exerts anticoagulant, antiplatelet, and fibrinolytic properties. Damage to the endothelium and the resultant reduction in the bioavailability of nitric oxide contributes to the initiation and progression of atherosclerosis as well as associated complications [17]. These factors are all likely contributors to the higher incidence of adverse cardiovascular events seen in patients with a low or borderline ABI.

### Association of ankle-brachial index level with ischemic stroke

Generally, Ischemic stroke were divided into thrombotic, embolic, and systemic hypoperfusion type according to mechanisms. The prevalence of ischemic stroke reportedly increases with decreasing ABI [18, 19]. In addition, studies have shown that in the general patient population, those with a low ABI have a higher likelihood of future stroke events than those with normal ABI or borderline ABI [7]. In the present study, however, we found that patients with borderline ABI had a significantly higher incidence of stroke than those with a normal ABI. Moreover, the incidence of stroke among patients with a borderline ABI was similar to the incidence among those with a low ABI. We also found that although the prevalence of atrial fibrillation was similar in the three groups, the CHADs vasc score was significantly higher in patients with a low or borderline ABI than in those with a normal ABI. Likewise, the prevalence of arteriosclerotic risk factors such as hypertension, diabetes mellitus, and smoking increased with decreasing ABI. These risk factors likely account for the higher future stroke rate among patients with a borderline or low ABI.

### Future heart failure across ankle-brachial index category

In a community cohort of middle aged Americans, it was found that a low ABI was associated with a 40% higher risk of heart failure than a normal ABI, and a borderline ABI was also associated with an increased risk of heart failure [20]. Similarly, in the present study, we found that low and borderline ABIs were strongly associated with future heart failure in hospitalized cardiology patients. There are many factors, including arterial stiffness, that can contribute to the onset of heart failure [21]. Brachial ankle pulse wave velocity (baPWV) is measured as a surrogate marker of arterial stiffness, and we found that baPWV increased with decreasing ABI. Thus, increasing baPWV may be a key to explaining the association between decreasing ABI and the increased incidence of new-onset heart failure.

### Major bleeding risk and ankle-brachial index spectrum

There is little available data on the association between major bleeding risk and ABI, especially borderline ABI. Our data revealed that patients with a low ABI had an approximately 2-fold greater incidence of major bleeding than those with a borderline or normal ABI, and that the rates of major bleeding were similar in the borderline ABI and normal ABI groups. Cause of bleeding was previously showed that In addition, the patients with high HAS-BLED and high CHA_2_DS_2_-VASc were reportedly at high risk for bleeding [22]. Regarding antiplatelet and anticoagulation therapy, administration rate of antiplatelet drug such as aspirin, Thienopyridines, and cilostazol at discharge were significantly higher in the patients with low ABI than those of borderline and normal ABI. This was one of the reason why the patients with borderline ABI had higher risk of future stroke and similar risk of major bleeding compared with those with normal ABI. In daily practice, attention should be paid to the possibility of stroke especially in patients with borderline ABI.

### Clinical outcomes in patients without hemodialysis separated based on ABI category

Hemodialysis patients tend to have hard vessel wall because of their advancing atherosclerosis. Furthermore, they had higher adverse events compared with non-hemodialysis patients [23]. Therefore, we performed sub-analysis to evaluate incidence of future cardiovascular events across the ABI categories in non-hemodialysis patients. Our data clearly showed that incidence of future cardiovascular events was significantly increasing with decreasing ABI in non-hemodialysis patients.

### Limitations

This study has several limitations. First, this is single-center, retrospective study. Second, the study population was heterogeneous and special, because we enrolled consecutively hospitalized cardiology patients in whom ABI was measured. Third, we have no data regarding changes in lifestyle or medication during the observation period. Fourth, enroll period was widely, therefore, this might affect therapeutic intervention and outcome.

## Conclusions

We conclude that the long-term incidence of adverse events was markedly higher in patients with a low or borderline ABI than in patients with a normal ABI. We therefore suggest that more attention be paid to the borderline group, especially for the occurrence of cardiovascular death, stroke, and heart failure.

## Supporting information

S1 Table Cox proportional regression analysis of MACE in the non-hemodialysis patients. Multivariate Cox analysis also showed that not only low but also borderline ABI was an independent predictor of adverse cardiovascular events.
(DOCX)

S1 Excel Raw data of IMPACT ABI. Raw data of current study.
(XLSX)

## Abbreviations

ABI: ankle-brachial index
ACC/AHA: American College of Cardiology and the American Heart Association
eGFR: estimated glomerular filtration rate
IMPACT-ABI: Impressive Predictive Value of Ankle-Brachial Index for Very Long Term Outcome in Patients with Cardiovascular Disease
LVEF: left ventricular ejection fraction
MACE: major adverse cardiovascular events
MI: myocardial infarction
SBP: systolic blood pressure
