# Supplementary material for: Impressive predictive value of ankle-brachial index for very long-term outcomes in patients with cardiovascular disease: IMPACT-ABI study
Source: PLoS One. 2017 Jun 15;12(6):e0177609. doi: 10.1371/journal.pone.0177609 (PMC5472275; doi:10.1371/journal.pone.0177609)
Supplement: S1 Table — Multivariate Cox analysis also showed that not only low but also borderline ABI was an independent predictor of adverse cardiovascular events. (DOCX) [file pone.0177609.s001.docx]

Cox proportional regression analysis of MACE in the non-hemodialysis patients

| Variable | unadjusted HR | 95% CI | p-value | adjusted HR | 95% CI | p-value |
| --- | --- | --- | --- | --- | --- | --- |
| Age | 1.05 | 1.04-1.06 | <0.0001 | 1.05 | 1.03-1.06 | <0.0001 |
| Female gender | 0.65 | 0.47-0.88 | 0.006 | 0.68 | 0.48-0.98 | 0.038 |
| ABI ≤ 0.9 | 2.53 | 1.88-3.42 | <0.0001 | 1.77 | 1.28-2.44 | <0.0001 |
| 0.91 ≤ ABI ≤ 0.99 | 1.66 | 1.11-2.48 | 0.013 | 1.66 | 1.10-2.51 | 0.016 |
| Body mass index | 0.98 | 0.95-1.01 | 0.221 |  |  |  |
| Hb | 0.93 | 0.87-0.99 | 0.033 | 0.98 | 0.92-1.05 | 0.547 |
| UA | 0.99 | 0.99-1.01 | 0.793 |  |  |  |
| Previous HF | 2.86 | 2.04-4.02 | <0.0001 | 2.84 | 2.01-4.00 | <0.0001 |
| Previous MI | 1.47 | 1.07-2.03 | 0.018 | 1.23 | 0.89-1.70 | 0.213 |
| Previous stroke | 2.28 | 1.59-3.28 | <0.0001 | 1.63 | 1.16-2.43 | 0.007 |
| Previous Intra cranial bleeding | 1.31 | 0.49-3.51 | 0.597 |  |  |  |
| Creatinine | 1.58 | 1.13-2.20 | 0.007 | 0.99 | 0.98-1.02 | 0.752 |
| Atrial fibrillation | 1.58 | 1.13-2.20 | 0.007 | 1.27 | 0.90-1.80 | 0.176 |
| Hypertension | 1.29 | 0.99-1.68 | 0.063 |  |  |  |
| Smoking | 1.41 | 1.10-1.83 | 0.007 | 1.22 | 0.92-1.63 | 0.171 |
| Diabetes | 1.14 | 0.86-1.51 | 0.358 |  |  |  |
| Insulin user | 1.84 | 1.05-3.22 | 0.032 | 1.63 | 0.91-2.92 | 0.103 |
| Aspirins administration | 1.17 | 0.90-1.51 | 0.248 |  |  |  |
| Warfarins administration | 1.33 | 0.99-178 | 0.057 |  |  |  |

HR, hazard ratio; CI, confidence interval; ABI, ankle–brachial index; UA, uric acid; HF, heart failure; MI, myocardial infarction
